# Neuroprotective effects of *Chlorella vulgaris* loaded niosomes via SIRT1 activation in aluminum chloride-induced Alzheimer’s model

**DOI:** 10.1038/s41598-025-25892-7

**Published:** 2025-11-18

**Authors:** Rania A. Radi, Mohamed A. Kandeil, Eman T. Mohammed, Marwa A. Ibrahim, Amr Gamal, Abdel-Razik H. Abdel-Razik, Fatma Khalil, Dina Sabry

**Affiliations:** 1 Department of Biochemistry, Faculty of Veterinary Medicine, Beni-Suef University, Beni-Suef, 62511 Egypt; 2 Department of Biochemistry and Molecular Biology, Faculty of Veterinary Medicine, Cairo University, Giza, 12211 Egypt; 3 Department of Pharmaceutics and Industrial Pharmacy, Faculty of Pharmacy, Beni-Suef University, Beni-Suef, Egypt; 4 Department of Histopathology, Faculty of Veterinary Medicine, Beni-Suef University, Beni-Suef, 62511 Egypt; 5 Animal and Poultry Management and Wealth Development Department, Faculty of Veterinary Medicine, Beni-Suef University, Beni-Suef, 62511 Egypt; 6 Department of Medical Biochemistry and Molecular Biology, Faculty of Medicine, Badr University in Cairo, Cairo, 11829 Egypt; 7 Department of Medical Biochemistry and Molecular Biology, Faculty of Medicine, Cairo University, Cairo, 11562 Egypt

**Keywords:** AlCl_3_, Alzheimer’s disease, *Chlorella vulgaris* Niosome, SIRT1, miRNA-134, Biochemistry, Drug discovery, Neurology, Neuroscience

## Abstract

Aluminum exposure is linked to the development of many neurodegenerative disorders including Alzheimer’s disease (AD), by disrupting molecular and cellular homeostasis in the brain. *Chlorella vulgaris* (CV), a green microalga, is reported to have antioxidant, anti-inflammatory, and neuroprotective properties. However, their role on Aluminum chloride (AlCl_3_)-induced AD and amyloid β (Aβ) clearance has not yet been fully elucidated. This research aimed to investigate the protective effectiveness of CV-loaded niosome (CV-LN) as a drug delivery system, in comparison to free form, against AlCl3-induced Alzheimer’s-like neurodegeneration in rats with special emphasis on SIRT1/miRNA-134/GSK3β axis. A niosomal formulation of Span 60, and cholesterol was chosen as an optimum formulation. Administration of CV or CV-LN dramatically improves the impaired oxidative markers, behavioral deficits, cholinergic and serotonergic dysfunctions, by significantly inhibiting monoamine oxidase, acetylcholinesterase activities and increasing serotonin level in brain of AlCl_3_-exposed rats. In parallel, CV or CV-LN triggers the activation of Sirtuin-1 (SIRT1) which downregulates miRNA-134, leading to increased brain-derived neurotrophic factor (BDNF) expression and improved neuronal survival and synaptic plasticity. Furthermore, CV or CV-LN decreases glycogen synthase kinase 3 beta (GSK3β)-mediated Tau hyperphosphorylation associated with clearance of Aβ. Additionally, CV or CV-LN significantly inhibits neuroinflammation by decreasing glial fibrillary acidic protein (GFAP), and apoptosis via modulating BAX, Caspase-3 and BCL2. Histopathological evaluations also supported the above findings. CV-LN formulations exhibited greater neuroprotective efficacy in a rat model, possibly due to better brain delivery and bioavailability. Eventually, CV and particularly CV-LN may hold promise as potential therapeutic candidates for further investigation in the context of neurodegenerative disorders and AD, possibly through modulation of SIRT1/miRNA-134/GSK3β axis.

**Supplementary Information:**

The online version contains supplementary material available at 10.1038/s41598-025-25892-7.

## Introduction

Aluminum (Al) toxicity has become a major concern owing to its possible involvement in neurodegenerative disorders, especially Alzheimer’s disease (AD). Chronic aluminum exposure disrupts the molecular and cellular equilibrium in the brain, thereby facilitating the onset and progression of AD. Understanding these pathways is crucial for designing treatments that prevent or reduce aluminum-induced neurodegeneration in AD. Aluminum can accumulate in the human body through various sources, including water, foods, toothpaste, and pharmaceutical products^1^. Once absorbed, Al has the ability to cross the blood–brain barrier and accumulate in the brain. It alters brain chemistry, damages DNA, and inhibits antioxidant enzymes^2^. Prior studies have linked Al neurotoxicity to oxidative stress, mitochondrial dysfunction, neurotransmission disruption, amyloid-beta (Aβ) plaque accumulation, hyperphosphorylation of Tau proteins (p-Tau), and development of neurofibrillary tangles (NFTs). Further amyloid-β protein accumulates in the AD brain and degenerates neurons^3^. These processes work together to cause synaptic disruption, neuronal death, and cognitive decline, suggesting aluminum’s possible contribution to the development of AD. The pathophysiology of AD includes intricate mechanisms and disturbances of the neuronal cascade involved in memory function. The early onset of AD has been identified in those under 65. However, about 90% of identified instances of AD are late-onset, and this typically affects individuals over 65^4^. Researchers are paying close attention to the theory that inflammation and oxidative stress have a role in the development of AlCl_3_-induced AD. Accordingly, the use of natural supplements with anti-inflammatory and antioxidant qualities could prevent or delay the onset of AD^5^.

*Chlorella vulgaris* (CV) is a unicellular green microalga that is highly enriched with nutrients like protein (45%), fat (20%), carbohydrates (20%), minerals (10%) (Such as magnesium and zinc), vitamins, and fibers (5%). Beyond its nutritional benefits, C. vulgaris is valued for its bioactive components like vitamins C and E, polyphenols, omega-3 and omega-6 fatty acids, lutein, lycopene, astaxanthin and other carotenoids^6^. These bioactive substances work synergistically to contribute to its powerful antioxidant, anti-inflammatory, and neuroprotective effects^7^. Research highlights the wide range of medicinal uses of CV in lowering blood sugar and cholesterol, regulating immunity, providing cardioprotective advantages^6,7^, and even reducing pro-apoptotic proteins in fish intestines^8^.

Niosomes and liposomes are examples of nanoparticles that could be used to deliver drugs in a more precise and regulated way^9,10^. Niosomes are a type of nanoparticles which composed of bilayers of non-ionic surface-active agents which are usually stabilized by addition of cholesterol^11^. They enhance the targeting, bioavailability, and sustainability of medications and are chemically stable, biodegradable, and biocompatible. They also exhibit low toxicity^11,12^. Niosomes are better than liposomes since phospholipids are readily hydrolyzed and oxidized^9,13^.

While the antioxidant properties of CV are recognized, its neuroprotective role on AlCl3-induced AD pathogenesis remains largely uninvestigated. Besides, the rigid, chitin-rich cell wall of CV restricts the release and bioavailability of its bioactive substances^14^, making its therapeutic application challenging. Therefore, the present study aimed to develop, characterize and explore the neuroprotective potential of CV-loaded niosome formulation, compared to free CV, against AlCl₃-induced Alzheimer’s-like pathology, with a specific focus on the SIRT1/miR-134/GSK3β axis and amyloid-β clearance. This study represents the first attempt, to our knowledge, to apply a nano formulated version of *Chlorella vulgaris* (CV-LN) as a drug delivery approach against neurodegeneration, offering a novel strategy to enhance bioavailability and therapeutic efficacy in neurodegenerative disease models.

## Materials and methods

### Chemicals

Aluminum chloride hexahydrate (AlCl₃·6H₂O; MW 241.43 g/mol; purity ≥ 98%; CAS No. 7784–13-6) was procured from Qualikems Fine Chem Pvt. Ltd. (Vadodara, India), while cholesterol, Span 60, dihexadecyl phosphate, methanol, and chloroform were purchased from Agitech Pharmaceutical Company (Cairo, Egypt). *Chlorella vulgaris* (CV) was produced as a pure green powder from Algal Biotechnology Unit (*National Research Centre, Dokki, Giza, Egypt*). Additional chemicals included in this experiment were purchased from *Sigma-Aldrich in St. Louis, Missouri, in the United States*.

### Preparation of CV-loaded Niosomes

Algae-loaded niosomes formulation was prepared using thin film hydration method as described by Guinedi, et al.^15^. 30 mg of span 60 and cholesterol were dissolved in a round flask with a molar ratio of 1:1 using a solution of methanol and chloroform. After that, the entire mixture was rotary evaporated at 40 °C with decreased pressure until a thin film was created. After drying, the thin film was hydrated for two hours at 60 °C using 10 ml of phosphate buffer with a pH of 7.4 and 10 mg of CV algae. To separate the entrapped and un-entrapped drugs, the produced formulation was sonicated for 30 min and then centrifuged using a cooling centrifuge set at 15,000 rpm and 4 °C for 1 h. The collected niosome pellets were again suspended in the phosphate buffer, and the resulting formulation was kept for further study at 4 °C.

### In vitro* characterization of CV-loaded Niosomes*

Zeta potential, particle size, and polydispersity index (PDI) of the synthesized niosome formulations were evaluated using the Malvern PCS4700 Instruments^16^. Prior to measurement, a sample of the CV-loaded niosome formulation was diluted with de-ionized water in order to provide the proper scattering intensity. Three independent measurements were taken at the room temperature. Morphological analysis was conducted using Transmission Electron Microscopy (TEM)^16^. For TEM visualization, a drop of a 1% aqueous solution of phosphotungstic acid dye was applied to the freshly prepared sample, which was then placed on top of a carbon-coated copper grid. Images were captured at appropriate magnifications. Differential Scanning Calorimetry (DSC) equipped with a liquid nitrogen chilling system was used to determine the thermal pattern and compatibility of the CV-loaded Niosome formulation with its constituents^17^. DSC thermograms were conducted for CV, Span 60, cholesterol and CV loaded niosomes. Heating was conducted from room temperature to 200 °C at a rate of 5 °C/min under a nitrogen flow of 25 mL/min. The chemical interactions of CV, cholesterol, span 60, and CV loaded niosomes were analyzed via Fourier Transform Infrared Spectroscopy (FTIR) (8400s, Shimadzu, Japan)^17^ using KBr pellet method, with spectra recorded in the 4000–400 cm⁻^1^ range.

### Animals

We obtained twenty-eight mature male albino rats from *Egypt’s Helwan Research Animal Farm*, weighing between 120 and 150 g. The rats were habituated for two weeks prior to being grouped in well-ventilated metal cages under a 12-h light/dark cycle at room temperature (24 ± 2 °C) and 68% humidity. Throughout the experiment, the rats had unrestricted access to both water and food. All experimental methods were approved by *Beni-Suef University’s Institutional Animal Care and Use Committee* and were conducted strictly in accordance with the guidelines for the care and use of laboratory animals. All experiments with animals complied with the ARRIVE (Animal Research: Reporting of In Vivo Experiments) guidelines (https://arriveguidelines.org). The procedures were approved under permission number 024–017.

### Experimental design

#### Induction of AlCl_3_-induced Alzheimer’s-Like neurodegeneration

The AlCl_3_-Induced Alzheimer’s-Like Neurodegeneration was induced by dissolving hydrated AlCl_3_ (AlCl_3_.H_2_O) in distilled water, and the animals were given 0.5 mL of the AlCl_3_.H_2_O solution orally for sixty days at a daily dosage of 100 mg/kg^18^. This dose was selected based on prior evidence demonstrating its ability to reliably induce neurotoxic and cognitive impairments. The rats were randomly divided into four equal groups (n = 7/ group) as follow:

- Normal control group: The rats were given oral gavages of distilled water only (vehicle) at a rate of 0.5 ml/kg b.w. per day.
- AlCl_3_ group: The rats were orally gavaged with 0.5ml freshly prepared hydrated aluminum chloride miscible in distilled water (100 mg/kg b. w./day) for 60 days.
- CV/AlCl_3_ group: The rats received a daily oral dose of freshly dissolved CV in D.W. at a dose of 100 mg/ kg b. w. before AlCl_3_ administration by an hour^19^. The CV dose was selected based on previous studies that reported significant antioxidant effects at this concentration^19^.
- CV-LN/AlCl_3_ group: The rats were given an oral gavage every day of CV-LN (100 mg/kg b. w.) before AlCl_3_ administration by an hour^19^.

#### Behavioral tests

At the end of the experiment, the animals underwent behavioral studies using the Y-maze test and Novel object recognition to investigate the learning, memory and cognition capability in rodents. Five rats per group were used. Behavioral tests were conducted and analyzed in a blinded manner.

#### y-maze test

By recording the spontaneous alternative behavior in the maze arms, the test evaluates spatial short-term working memory^20^.

#### Novel object recognition (NOR)

Novel Object Recognition (NOR) aims to evaluate the short-term memory. According to the protocol selected by Bevins and Besheer^21^, Leger et al.^22^, and Lim et al.^23^, rats naturally engage with new items more than they do with old ones, a phenomenon known as recognition memory, which is the basis for the novel object recognition test, for evaluating the learning and memory deficiencies in rats and mice. NOR was assessed using the discriminating index (DI), and discriminating Ratio (DR).

$${\text{DI = }}\frac{{\text{Time exploring novel object}}}{{\text{Total time exploring both novel and familiar objects}}}$$

$${\text{DR = }}\frac{{\text{Number of attempts to explore novel object}}}{{\text{Total Number of attempts to explore both novel and familiar objects}}}$$

#### Sampling and tissue preparations

At the end of the experiment, animals were humanely euthanized using cervical dislocation, the procedure was performed quickly and efficiently to minimize any potential pain or distress. An incision was made on the dorsal side of the skull, and the brains were collected, cleaned, and washed with physiological saline (0.9% sodium chloride). The cortex and hippocampus were removed from one hemisphere then divided into 2 parts. Using a homogenizer (Ortoalresa, Spain), the first piece was homogenized in 5ml of phosphate buffer saline (pH: 7). Then, it was centrifuged at 20,000 ×*g* for 15 min at 4 °C, and the supernatant was stored at − 20 °C for additional biochemical tests of reduced glutathione (GSH), Malondialdehyde (MDA), and Catalase activity. For molecular analysis, the second piece of brain tissue was kept at − 80 °C. The other hemisphere was cleaned in saline, then immersed in 10% neutral buffered formalin for histological analysis.

### Biochemical evaluations

#### Determination of oxidative stress biomarkers in brain tissue

Commercial assay kits for Catalase activity, MDA, and GSH were obtained from the *Biodiagnostic Company for Research Kits (Egypt)*, and used for the measurements within the tissue of the brain homogenates using the techniques outlined by Aebi^24^, Satoh^25^, and Beutler and Kelly^26^, respectively.

#### Determination of Rat Amyloid Beta, BDNF, GFAP, GSK3β. SIRT1 and serotonin contents in brain tissue

Determination of Amyloid β-protein (Aβ1-42) levels was estimated using the required ELISA kit according to the protocols provided by the manufacturers (*Biorbyt, LLC. San Francisco, California *94,104,* USA*). BDNF concentrations were determined using BDNF ELISA kit (*Biosensis, Pty Ltd., Thebarton 5031, SA, Australia*). GFAP concentrations were determined using GFAP ELISA kit (*Elabscience, Houston, Texas, *77,079,* USA*). *GSK3β* concentrations were determined using *GSK3β* ELISA kit (*FineTest, Wuhan, *430,074, *Hubei, China*). SIRT1 concentrations were determined using SIRT1 ELISA kit (*Bioss, Inc., Woburn, Massachusetts *01,801,* USA*). Serotonin concentrations were determined using Serotonin ELISA kit (*Enzo Life Sciences Inc., 10 Executive Boulevard, Farmingdale, NY *11,735). All assays were determined in the cortical and hippocampal tissues.

#### P-Tau by western immunoblotting

The ReadyPrep TM protein extraction kit (total protein) provided by Bio-Rad Inc (Catalog #163–2086) was employed according to manufacturer instructions for each sample of the homogenized brain tissue of all different groups. Bradford Protein Assay Kit (SK3041) provided by Bio basic Inc (Markham Ontario L3R 8T4 Canada) was used for quantitative protein analysis^27^. 20 μg of protein per lane were separated using 10% SDS-PAGE and then were loaded onto polyvinylidene difluoride membranes (PVDF). Using primary antibodies to p-Tau and β-actin, the membrane was examined as a loading control after being incubated for two hours with Tris-buffered saline (10 mM Tris–Cl, pH 7.5, 100 mM NaCl) containing 0.1% Tween 20 and 5% nonfat-dried milk. Developing and visualizing membranes by using of the Amersham detection kit and chemiluminescence in accordance with the manufacturer’s instructions. Image analysis software was used to read the band intensity of the target proteins against control sample beta actin (housekeeping protein as a reference control) by protein normalization on the ChemiDoc MP imager. We assessed p-Tau as triplicate samples which were normalized against triplicate housekeeping beta actin protein to ensure the validity of the results. We purchased the primary and secondary antibodies from Cell Signaling Technologies in the United States.

#### Assays of AChE, MAO, miRNA134, BAX, BCL2, and Caspase 3 genes expression

Making use of the TRIzol Reagent *(Life Technologies, USA*) and Direct-zol RNA Miniprep Plus (*Cat# R2072, ZYMO RESEARCH CORP. USA*), RNA was extracted from tissue lysate in accordance with the manufacturer’s instructions. The SuperScript IV One-Step RT-PCR kit (*Cat# *12,594,100*, Thermo Fisher Scientific, Waltham, MA USA*) was used to reverse-transcribe the extracted RNA before a single-step PCR was carried out. The Table 1 contains a list of the gene-specific primers. The NCBI platform was used to design the primers. The thermal cycling protocol consisted of 40 cycles, each comprising denaturation at 95 °C for 10 s, annealing at 58 °C for 15 s, and extension at 72 °C for 15 seconds^28^. The specificity was then confirmed using a melting curve analysis. The delta-delta Ct (ΔΔCt) was used to measure each gene expression’s relative quantification (RQ), with U6 serving as the housekeeping gene for miRNA-134 and β-actin used for normalization of other mRNA genes^29^. No experiment included template controls for any of the genes. Every sample underwent two analyses^30^. The delta-delta Ct (ΔΔCt) calculation is used to determine the relative quantification of each target gene. We calculated the RQ for each gene using 2-∆∆Ct^31^.

**Table 1:** The primer sequences for the assessed genes.

Gene	Primer sequence (5′ → 3′)	Accession no
AChE	AGGACGAGGGCTCCTACTTT-CATGGCATCTCTCAGGTGGG	NM_172009.1^32^
MAO	GTGCCTGGTCTGCTCAAGAT-GGCCCAAACCATAGGCTGTA	NM_033653.1^33^
miRNA-134	GGGTGTGACTGGTTGACC-CAGTGCGTGTCGTGGAGT	MI0000907^34^
U6 (reference gene)	GCTTCGGCAGCACATATACTAAAAT-CGCTTCACGAATTTGCGTGTCAT	XR_010061656.1^34^
BCL-2	TCGCGACTTTGCAGAGATGT-CAATCCTCCCCCAGTTCACC	NM_016993.2^35^
BAX	CACGTCTGCGGGGAGTCAC-TTCTTGGTGGATGCGTCCTG	NM_017059.2^36^
Casp3	GAGCTTGGAACGCGAAGAAA-TTGCGAGCTGACATTCCAGT	NM_012922.2^37^
β-Actin (reference gene)	CCGCGAGTACAACCTTCTTG-CAGTTGGTGACAATGCCGTG	NM_031144.3^38^

#### Histopathological studies

The cortex and hippocampus of one hemisphere were removed, washed with saline, and then submerged in 10% neutral buffered formalin. After being dehydrated by immersing the tissue samples in gradient concentrations of ethanol, they were sectioned using a rotating microtome to a thickness of 4–6 μm, washed and cleared in xylene, impregnated in soft paraffin, embedded in hard paraffin, and put on clear, dry glass slides. Tissue is examined using a variety of histopathological stains, such as Luxol Fast Blue for myelin sheath identification, Congo red stain for amyloid detection in brain tissue, and Hematoxylin and Eosin as a general stain. The obtained slides underwent examination and observation using a light microscope attached to a LEICA (DFC290 HD system digital camera, Heerbrugg, Switzerland) employing 10, 20, and 40 objective lenses^39^. Histopathological examinations were conducted in a blinded manner for all treated groups.

#### Statistical analysis

The findings were statistically analyzed using SPSS (version 27.0.1). After the one-way analysis of variance (ANOVA) test, the Tukey’s post hoc test was used to compare the experimental groups. The results were presented as the mean with standard error of the mean and were statistically significant at the P < 0.05 level.

## Results

### In vitro characterization of CV-loaded niosomes

#### Preparation of CV-loaded niosomes

According to a review of the literature and early research, the ideal concentration of cholesterol and non-ionic surfactants to form niosomes with the right consistency for Tiamulin distribution was 30 mg. It was discovered that adding more cholesterol and non-ionic surfactants increased the hydrophobic domain’s volume and the number of niosomes that formed, which increased the particle size and entrapment efficiency. The development of mixed micelles alongside niosomal vesicles may be the cause of the decrease in entrapment effectiveness and increase in particle size seen when these components were increased over 30 mg. At comparable molar ratios with cholesterol, Span 60 produced lower particle sizes than Tween 60 among the tested surfactants. Consequently, 30 mg of Span 60 and cholesterol in a 1:1 molar ratio were successfully used to create CV-loaded niosomes.

#### Zeta potential, particle size, and size distribution

**Fig. 1:**
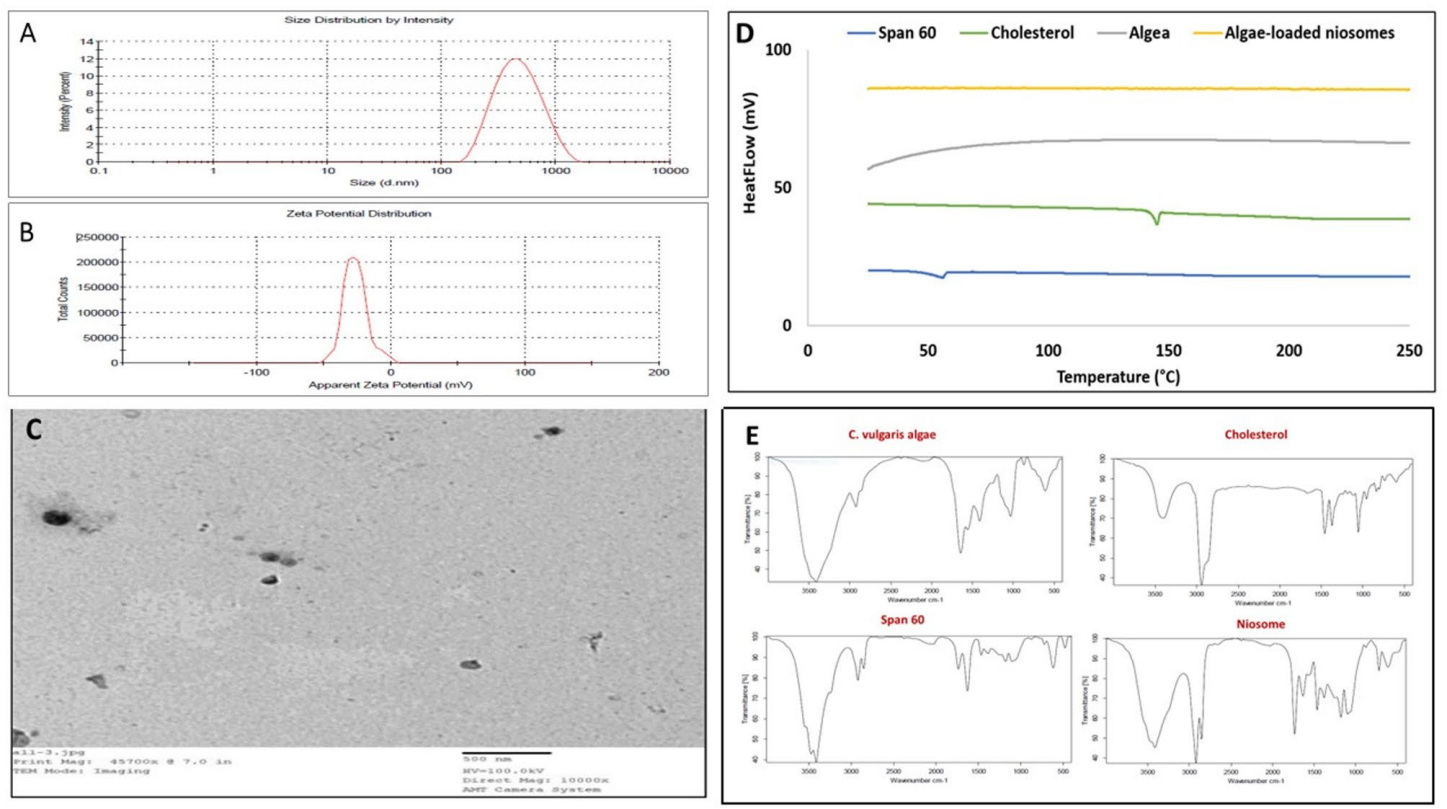
(**A**) Vesicle size, (**B**) zeta potential of CV-loaded niosomes, (**C**) TEM image of CV-loaded niosomes, (**D**) DSC thermograms of CV-loaded niosomes, (**E**) FTIR spectrums for the CV, cholesterol, Span 60, and CV-loaded niosomes. FTIR: Fourier-transform infrared spectroscopy. TEM: Transmission Electron Microscopy, DSC: Differential Scanning Calorimetry.

A small particle size of 367.3 d.nm was observed in the formulation of CV-loaded niosomes, indicating the creation of nano-sized vesicles. A limited size distribution and good homogeneity were indicated by the polydispersity index (PDI), which was determined to be 0.262 (Fig. 1A). Furthermore, zeta potential studies showed a negative surface charge of  − 26.7 mV, which is sufficient for electrostatic stabilization and indicates good physical and chemical stability (Fig. 1B).

#### Transmission electron microscopy (TEM)

Figure 1C showed a TEM microphotograph of CV loaded niosomes, which appeared approximately spherical with smooth surface black dots. The niosomes are well dispersed without aggregation on the surface.

#### Differential scanning calorimetry (DSC)

The crystallinity is quantitatively estimated using the DSC. Figure 1D shows the thermal behavior of CV, Span 60, Cholesterol and CV-loaded niosomes formulation. The crystalline nature of CV was demonstrated by the prominent endothermic peak in the DSC thermogram of pure CV at its melting temperature of 25 °C. According to the thermal analysis, cholesterol showed an endothermic peak at 150 °C and Span 60 surfactant showed an endothermic peak at its melting point of 54.63 °C. The incorporation of CV into the niosomes, as demonstrated by the thermogram of the CV-loaded niosome formulation, reduced their crystallinity because the CV was incorporated as an amorphous form within the surfactant bilayer structure. The crystalline patterns of the Span 60 and cholesterol were lost as a result of their assembly as a bilayer in the formulated niosome, indicated by the disappearance of their peaks compared to those of cholesterol and Span 60, separately.

#### Fourier-transform infrared spectroscopy (FTIR)

Figure 1E displayed the FTIR spectrums of CV algae, Span 60, cholesterol, and CV loaded niosomes. The distinctive peaks in Span 60’s spectrum were located at 3413 cm^−1^ and 2920 cm^−1^, and they represent amines or the functional groups of hydroxyl and carboxylic acids, respectively. The alkyl substituted ether functional group is shown by the distinctive peak in the spectra of Span 60, which was located at 1179 cm^−1^. Additionally, the aldehyde functional group was indicated by the characteristic peak in the spectra of Span 60, which was located at 1628 cm^−1^. Peaks in the cholesterol FTIR spectrum were found at 3399 cm^ − 1^ for the OH stretching group, 2940 cm^ − 1^ for the CH2 stretching vibration, 1670 cm ^− 1^ for the double bond C = C, and 1459 cm^ − 1^ for the asymmetric CH2 stretching vibrations. The FTIR spectra of algae revealed peaks at 3415 cm^ − 1^ for the stretching of water (O–H) and protein (N–H), at 2927 cm^ − 1^ for the stretching of lipids and carbohydrates primarily (CH2) and (CH2), at 1644 cm ^− 1^ for the stretching of the protein amide I band primarily (C = O), and at 1033 cm^ − 1^ for the stretching of carbohydrates (C–O–C) of polysaccharides. Similar peaks in the CV-loaded niosomes’ FTIR spectra indicated that the formulation’s component elements were compatible.

### In vivo characterization of CV-loaded niosomes

#### Effect of CV or CV-LN on cognitive function in AlCl_3_-induced AD Rat Model

The changes in both spatial and non-spatial short-term memory (SAP%), as well as in memory indices (DI and DR), were depicted in Table 2. The SAP, DI, and DR values of untreated AlCl_3_-induced AD-like rats were significantly lower (p = 0.001) than those of the control group. Treatment with CV or CV-LN significantly improved the SAP scores, while the scores for DI and DR of rats treated with CV-LN were significantly (p = 0.001) higher than those of the AlCl_3_-induced AD-like animals. However, when compared to the free CV group, DI and DR were considerably higher (p = 0.003 and 0.001) in the CV-LN group, suggesting a better effect of niosomal formulation.

**Table 2:** Changes in cognitive function, cholinergic and monoaminergic markers in AlCl3-induced AD Rat Model.

Groups	SAP%	DI Novel object	DR Novel object	Gene expression levels		Serotonin (ng/mg protein)
				**AChE**	**MAO**	
Control	29.667 ± 1.016^a^	0.543 ± 0.053^a^	0.557 ± 0.053^a^	1 ± 0^a^	1 ± 0^a^	3.149 ± 0.074^a^
AlCl3	11.167 ± 0.737^b^	0.139 ± 0.017^b^	0.136 ± 0.017^b^	2.62 ± 0.03 ^b^	3.43 ± 0.08^b^	1.306 ± 0.022^b^
CV + AlCl3	23.167 ± 0.962^c^	0.268 ± 0.035^b^	0.139 ± 0.018^b^	1.65 ± 0.01 ^c^	1.78 ± 0.01^c^	2.065 ± 0.077^c^
CV-LN + AlCl3	22.333 ± 0.807^c^	0.471 ± 0.029^a^	0.614 ± 0.046^a^	1.54 ± 0.07 ^c^	1.89 ± 0.005^c^	2.757 ± 0.139^d^

Values are represented as mean ± standard error of mean (n = 7 for SAP%, DI, DR, n = 5 for AChE, MAO, serotonin). Columns with different superscript letters are significantly different at p < 0.05. AlCl3: Aluminum chloride, CV-LN: *Chlorella vulgaris*-loaded niosomes, SAP: Spontaneous alternation behavior, DI: Discrimination index, DR: Discrimination ratio. AChE: Acetylcholinesterase enzyme, MAO: Monoamine oxidase-A.

#### Effect of CV or CV-LN on cholinergic and monoaminergic markers in AlCl_3_-induced AD Rat Model

Table 2 presented that the AlCl_3_-induced AD-untreated rats had significantly higher levels of AChE and MAO mRNA expression and significantly lower levels of serotonin (p = 0.001) than the control group. The significant improvement of the values following treatment with CV or CV-LN formulations (p = 0.001) demonstrated an ameliorative effect. There were no significant variations between free CV or CV-LN treatment groups in the expression levels of both AChE and MAO enzymes. While the treatment with niosomal formulation resulted in a better effect on serotonin levels when compared to the free CV group.

#### Effect of CV or CV-LN on brain redox markers in AlCl_3_-induced AD Rat Model

**Fig. 2:**
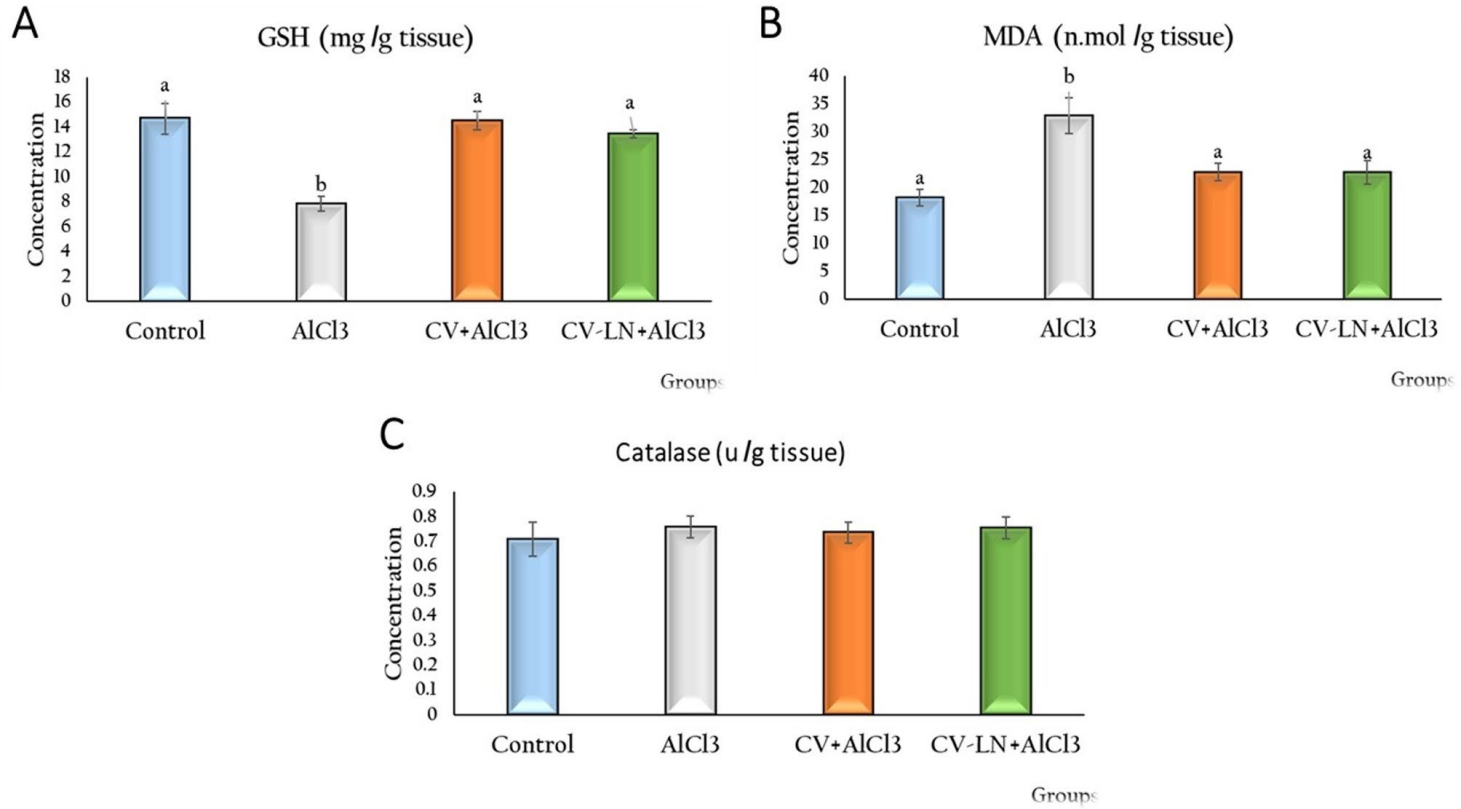
Effect of CV or CV-LN on (**A**) GSH (**B**) MDA and (**C**) Catalase activity in brain of AlCl_3_ exposed rat. Values are represented as mean ± standard error of mean (n = 7). Columns with different superscript letters are significantly different at p < 0.05.

In contrast to control rats, the AlCl_3_-induced AD-like untreated rats exhibited a significant decrease (p = 0.001) in GSH and a significant increase (p = 0.001) in MDA concentrations, as shown in Fig. 2. By bringing the results back to almost normal, treatment with CV or CV-LN formulations clearly demonstrated an ameliorative effect. However, there were no appreciable differences in GSH, MDA, or Catalase levels between the CV and its nano-formulation (CV-LN) groups. There was no significant difference in the catalase enzyme activity in brain tissue homogenates between the various rat groups.

#### Effect of CV or CV-LN on brain Beta-amyloid, p-Tau, GSK3β, GFAP, SIRT1, BDNF and miRNA-134 concentrations in AlCl_3_-induced AD Rat Model

**Fig. 3:**
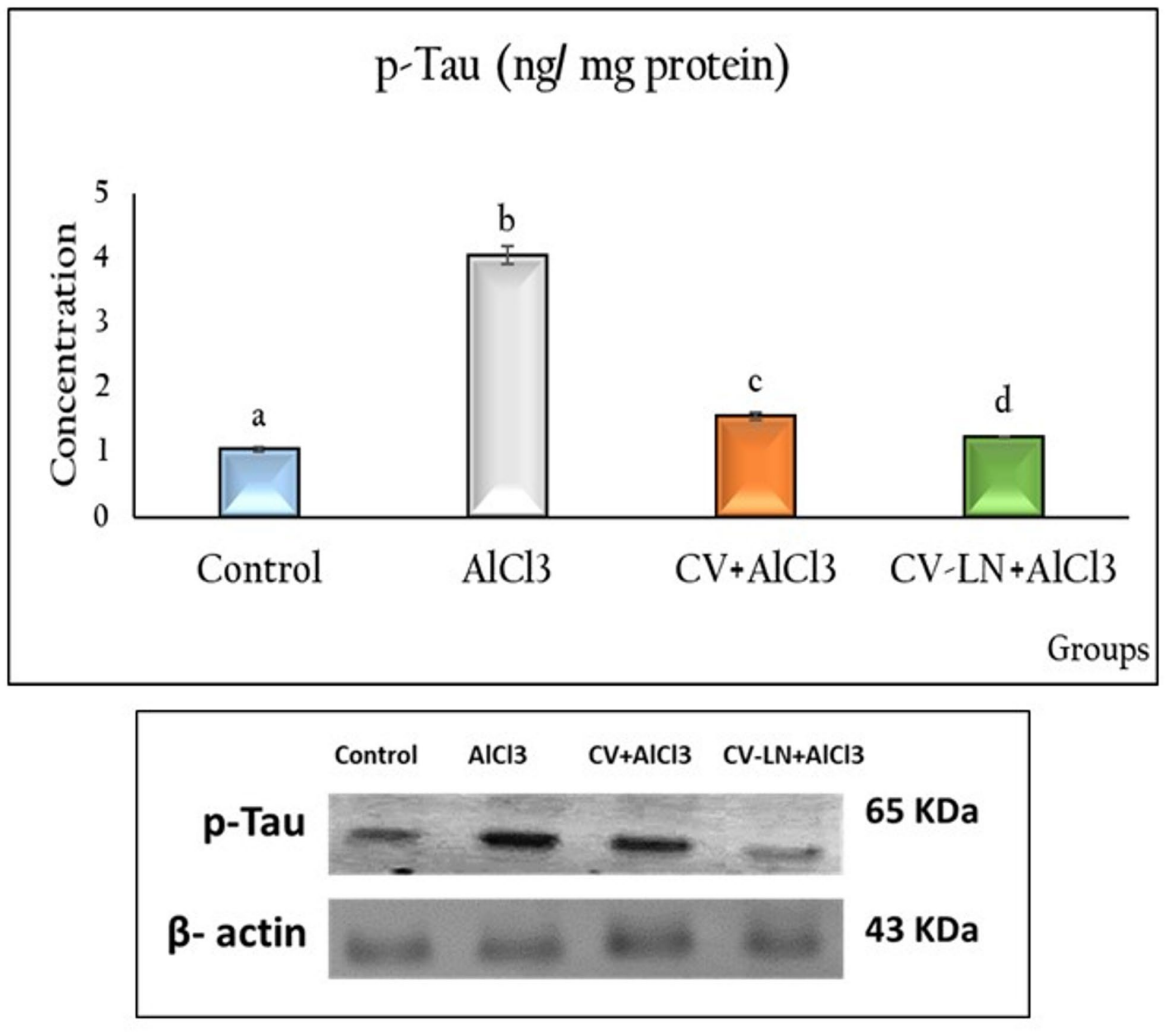
Effect of CV or CV-LN on P-Tau concentrations in brain of AlCl_3_ exposed rat. Values are represented as mean ± standard error of mean (n = 5). Columns with different superscript letters are significantly different at p < 0.05.

Table 3 and Fig. 3 showed that AlCl_3_-induced AD-like untreated group had significant higher (p = 0.001) levels of Aβ1-42, p-Tau, GSK3β, and GFAP, as well as a higher expression of miRNA-134 and significant lower levels of SIRT1 and BDNF (p = 0.001) in brain tissues as opposed to the control group. Treatment with CV or CV-LN formulations demonstrated an ameliorative effect indicated by the significant (p = 0.001) improvement in the values when compared to the AlCl_3_-induced AD group. However, as compared to the free-form, the nano-formulations demonstrated the strongest protection for all tested markers except for GFAP and miRNA-134 levels which did not change between both groups.

**Table 3:** Changes in Beta-amyloid, *GSK3β*, GFAP, SIRT1, BDNF and miRNA-134 concentrations in the brain of AlCl_3_-induced AD Rat Model.

Groups	Beta-Amyloid (Aβ1-42) (pg/mg protein)	GSK3β (ng/mg protein)	GFAP (ng/mg protein)	SIRT1 (ng/mg protein)	BDNF (pg/mg protein)	miRNA-134 (ng/mg protein)
Control	1.257 ± 0.049^a^	1.118 ± 0.060^a^	2.842 ± 0.179^a^	2.425 ± 0.042^a^	154.0 ± 2.39^a^	1.170 ± 0.049^a^
AlCl3	7.481 ± 0.212^b^	2.959 ± 0.065^b^	9.923 ± 0.539^b^	0.581 ± 0.005^b^	28.01 ± 1.28^b^	5.323 ± 0.136^b^
CV + AlCl3	3.756 ± 0.112^c^	1.976 ± 0.049^c^	3.103 ± 0.217^a^	1.234 ± 0.029^c^	62.33 ± 2.61^c^	1.411 ± 0.0104^a^
CV-LN + AlCl3	2.579 ± 0.119^d^	1.216 ± 0.031^a^	3.016 ± 0.122^a^	2.114 ± 0.099^d^	125.53 ± 2.45^d^	1.136 ± 0.035^a^

Values are represented as mean ± standard error of mean (n = 5). Columns with different superscript letters are significantly different at p < 0.01. AlCl_3_: Aluminum chloride, CV-LN: *Chlorella vulgaris*-loaded niosomes, GSK3β: Glycogen synthase kinase-3 beta, GFAP: Glial Fibrillary Acidic Protein, SIRT1: Rat Sirtuin 1, BDNF: Brain derived neurotrophic factor, miRNA: Micro Ribonucleic acid.

#### Effect of CV or CV-LN on brain apoptotic markers in AlCl_3_-induced AD Rat Model

**Fig. 4:**
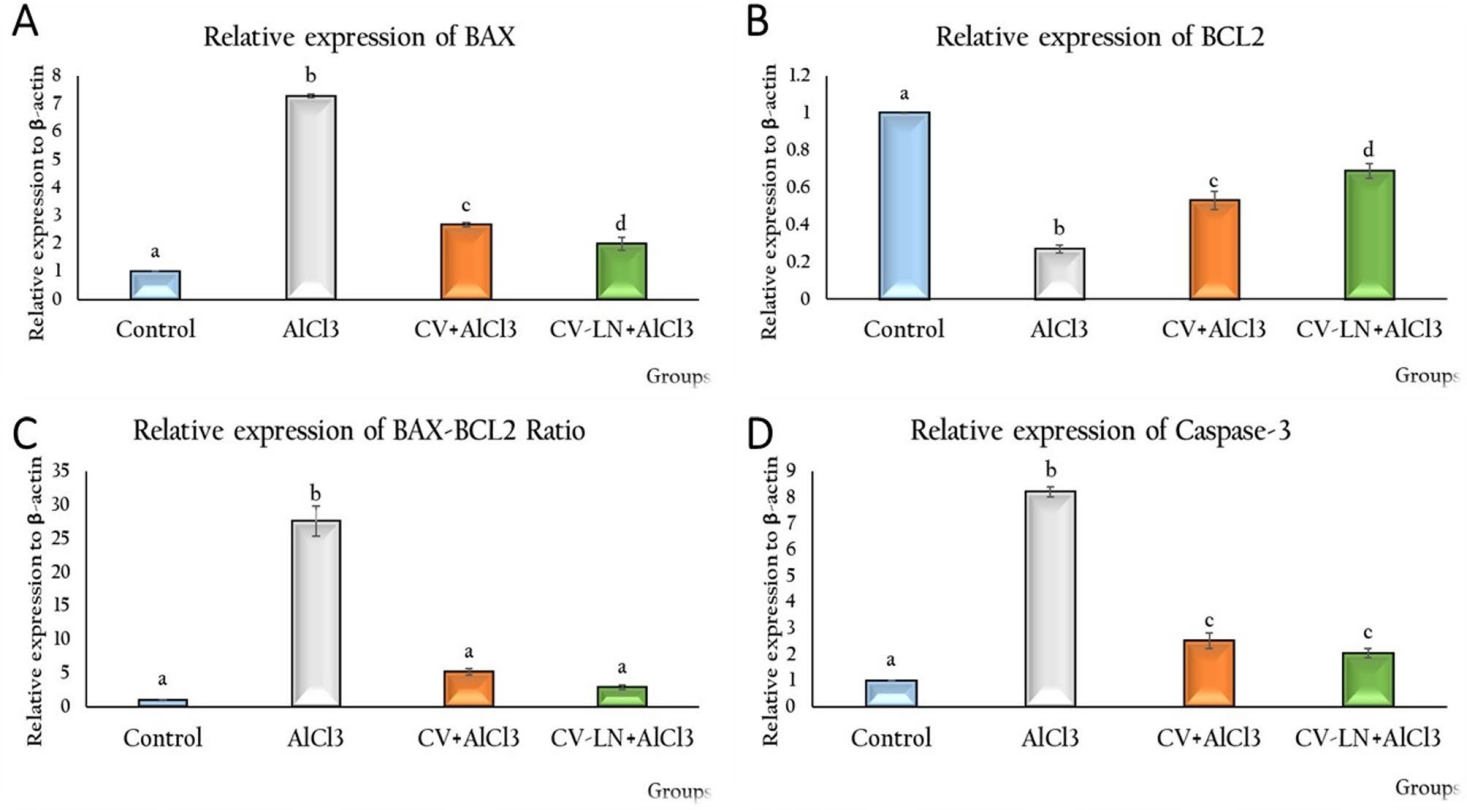
Effect of CV or CV-LN on (**A**) BAX (**B**) BCL2 (**C**) BAX-BCL2 ratio and (**D**) Caspase-3 activity in brain of AlCl_3_ exposed rat. Values are represented as mean ± standard error of mean (n = 5). Columns with different superscript letters are significantly different at p ≤ 0.01.

The results in Fig. 4 showed the alterations in mRNA relative expressions of BAX, BCL2, BAX/BCL2 ratio and Caspase-3 in the brain tissues. Compared to the normal control group, the AlCl_3_-induced AD-untreated group exhibited significantly (p = 0.001) lower levels of BCL2 and significantly (p = 0.001) greater levels of BAX, the BAX/BCL2 ratio, and Caspase-3. Treatment with CV or CV-LN showed an ameliorative effect (p = 0.001) compared to the AlCl_3_-induced AD group. The niosomal formulations produced the best results of BAX (p = 0.006) and BCL2 (p = 0. 011) compared to the free form.

#### Effect of CV or CV-LN on histopathological structure in the brain of AlCl_3_-induced AD Rat Model

**Fig. 5:**
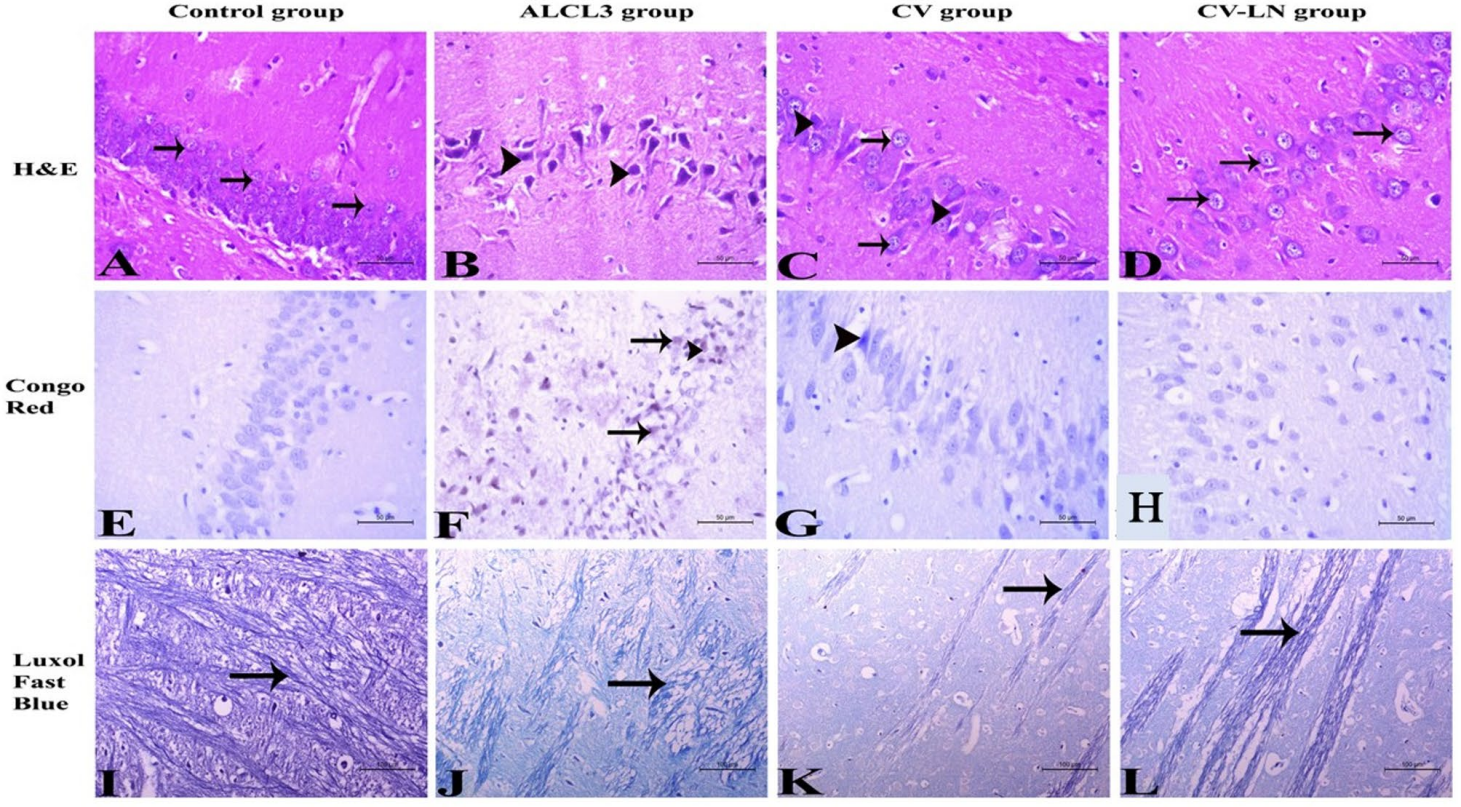
Histopathological changes of the hippocampus in different studied groups. (**A**): Hippocampus of the normal control group showing normal hippocampal tissue with several layers of normal neurons (arrow) and neuroglia cells. (**B**): Brain tissues of AlCl3- rats showing the hippocampus appeared with disoriented cell layers. The nerve cells and neuroglia cells possessed severe degenerative changes and shrinkage (arrowhead). (**C**): Brain tissues of CV group showed that the hippocampus was regaining its layers architecture. The majority of nerve cells and neuroglial tissue (arrow) appeared normal while few neuroglia cells showed mild degeneration (arrowhead). (**D**): Brain tissues of CV-LN formulation group possessed normal orientation of the hippocampus with normal neurons and normal neuroglia cells (arrow). *H&E stain X400*. (**E**): Hippocampus of the normal control group didn’t show any foreign materials. (**F**): Brain tissues of AlCl3 exposed rats showing deposition of amyloid materials in the hippocampal tissue (arrow) and inside the nerve cells and/or neuroglia cells (arrowhead). (**G**): Brain tissues of CV group show acidophilic amyloid materials inside the neurons and/or neuroglia cells (arrowhead). (**H**): Brain tissues of CV-LN formulation group didn’t show any deposition of foreign materials. *Congo red stain X400*. (**I**): Brain tissues of the normal control group showing long nerve fibers with continuous myelin sheath. **J**: Brain tissues of AlCl3 exposed rats showing the majority of nerve fibers appeared with irregular and ill myelination. **K**: Brain tissues of CV group showing nerve fibers with irregular myelin sheath. (**L**): Brain tissues of CV-LN formulation group with long nerve fibers with continuous myelin sheath. *Luxol Fast Blue stain X200.*

Figures 5B–J illustrated how the brain tissues of the AlCl_3_-induced AD-like untreated group displayed disrupted cell layers in the hippocampus when compared with those of control (Fig. 5A–I). Significant degenerative alterations and shrinking were observed in the nerve and neuroglia cells. Amyloid material accumulation was observed in the hippocampus, inside nerve cells and/or neuroglia cells, and most nerve fibers showed signs of inadequate myelination and irregularity. As illustrated in Fig. 5C–L, treatment with CV or CV-LN formulations greatly improved the condition by the significant recovery of the hippocampus tissue. The greatest results, however, were obtained with CV-loaded niosome formulations.

## Discussion

CV-loaded niosomes were successfully formulated with 30 mg of Span 60 and cholesterol in a 1:1 molar ratio, to investigate their role as a drug delivery system. There are multiple reasons for the observed variations in particle size between the Tween 60 and Span 60 formulations. Span 60 has a larger hydrophobic moiety, a lower hydrophilic-lipophilic balance (HLB) value, and longer C-H alkyl chains^40^. Furthermore, Span 60’s lower surface free energy and greater gel-liquid phase transition temperature help to create smaller, more stable vesicles^41^. Additionally, it was shown that cholesterol was essential for preserving the rigidity, stability, and integrity of vesicles^42^. When the cholesterol concentration was raised above the equimolar ratio with Span 60, the bilayer membrane became stiffer, which led to the creation of bigger vesicles^17^. However, too much cholesterol could reduce the effectiveness of drug entrapment by competing with the drug for space within the bilayer^43^. A molar ratio 1:1 of Span 60 to cholesterol is thought to be ideal for effective drug administration through niosomes, according to the literature ^41,43^. Because smaller vesicles have a bigger surface area and a better potential for drug absorption, vesicle size is a critical factor in determining the effectiveness of niosomes ^44,45^. The current formulation’s nanoscale size indicates a promising medication delivery profile. For reliable drug release and distribution, size uniformity is necessary, as indicated by a low PDI value of 0.262. The formulation’s sufficient electrostatic repulsion between vesicles, which promotes colloidal stability and lessens aggregation, is further demonstrated by the negative zeta potential of − 26.7 mV (Fig. 1A–B).

Growing evidence has shown that Aluminum is the most powerful neurotoxin. Because of its strong affinity for the receptors, Al can readily penetrate the blood–brain barrier and eventually accumulate in the brain, making it a powerful target for Al toxicity^2^. Numerous mechanisms have been demonstrated to contribute to AlCl_3_-induced neurodegeneration, including elevated expression of the amyloid precursor protein (APP), deposition of Aβ, formation of NFTs, oxidative stress, neuroinflammation, caspase activation, altered Akt/GSK3β signaling pathway in the cerebral cortex^46^, increased activity of acetylcholinesterase (AChE), modifications in neurotransmitters, impairment of hippocampal synaptic plasticity^47^, and loss of neurons^48^. It is commonly acknowledged that these degenerative alterations linked to AlCl_3_ underlie both cognitive and non-cognitive disorders that resemble those experienced by AD patients. In the current investigation, neurochemical changes in the cortex and hippocampus were linked to behavioral changes induced by AlCl_3_.

According to our findings, the AlCl_3_ group’s non-spatial working memory in the NOR test and spatial working memory in the Y-maze test were significantly reduced, as seen by the decline in the number of arm entries, which in turn led to a decline in the SAP%, DI%, and DR%. The SAP relies on the rats’ innate propensity to switch between the three limbs. Similar findings were obtained by Al exposure in the study of Anadozie et al.^49^ and Kandeil et al.^50^. In addition, Huang et al.^51^ claimed that there is a clear connection between high levels of aluminum and cognitive decline, especially in elderly. However, an increase in the SAP%, DI%, and DR% indicated a marked improvement in spatial working memory following CV or CV-LN therapy. These findings were consistent with other earlier research implied that consuming chlorella oil fraction enriched with docosahexaenoic acid efficiently improves working memory during maze performance^52^.

The disruption of cholinergic transmission is one of the main characteristics of AD which is intimately associated with cognitive decline. The enzyme acetylcholinesterase (AChE), which degrades acetylcholine (ACh), showed increased activity in the hippocampus and cortex of AlCl_3_-induced AD-like animals, interfering with cholinergic signaling. Aluminum exposure elevates AChE activity, accelerating ACh breakdown. Oxidative stress^53^ and Al toxicity^54^ further enhance AChE activity and damage the blood–brain barrier. This is linked to cognitive deficits and dementia, as supported by findings from Lin et al.^55^ and Hafez et al.^56^, who reported reduced ACh levels in AlCl_3_-induced AD in rats. A possible explanation for the increase in AlCl_3_-induced AChE activity is an allosteric interaction between aluminum ions and the anionic sites of the enzyme in the brain^57^. However, treatment with CV or CV-LN appears to counteract Al-induced increases in AChE activity, likely through its antioxidant effects. The polyphenol and carotenoid contents of microalgae are associated with their cholinesterase inhibitory actions. According to their protein composition, *Chlorella vulgaris* and *Spirulina platensis* aqueous protein extracts specifically suppressed AChE by roughly 20% and 46%, respectively^58^, demonstrating the potential of microalgal protein extracts as effective AChE inhibitors.

In the current investigation, AlCl_3_ increased MAO enzyme activity (Table 2) which is a mammalian flavoenzyme playing a critical role in the metabolism of the monoamine neurotransmitters like dopamine, serotonin, and norepinephrine. These findings are consistent with earlier research by Hafez et al.^56^ and Liu et al.^59^. It is currently unclear how exactly Al raises MAO; however, it may be due to oxidative stress, which can enhance MAO activity and expression^49^. A monoamine neurotransmitter, 5-hydroxytryptamine, also referred to as serotonin, is known to regulate a number of physiological processes, including mood, eating, emotions, sleep, and memory^60^. More specifically, AlCl_3_-induced neuroinflammation caused a complete decrease in monoamine serotonergic neurotransmitters, followed by an increase in AChE and MAO activity, which indicates neuronal injury. The present study found that AlCl_3_ intoxication significantly decreased serotonin levels, which is consistent with Kumar^61^. The present results demonstrated that the administration of CV or CV-LN may ameliorate the neurotransmitter balance by raising serotonin levels and decreasing AChE and MAO genes expressions, while also markedly raising the SAP, DI, and DR scores as compared to those in AlCl_3_-induced AD-like rats (Table 2).

One of the primary toxic mechanisms of aluminum is its capacity to induce oxidative brain damage which is also implicated in AD pathogenesis^3^. Because of the high demand of the brain for molecular oxygen to function properly, the accumulation of damaged biomolecules caused by ROS is high, especially with aging^62^. Aluminum exacerbates iron-induced lipid peroxidation by binding to transferrin receptors in the brain, which normally mediate iron transport^63^. By decreasing iron binding to transferrin, this interaction raises cell levels of free iron and encourages oxidative stress. This effect may also contribute to a transition to ferroptosis in PC12 cells subjected to Al exposure^64^.

The current research shows that the brains have high levels of MDA and low levels of GSH of AlCl_3_-induced AD-like rats (Fig. 2) are consistent with Lakshmi et al.^65^ and suggesting increased lipid peroxidation. These findings support the notion that oxidative stress and aberrant neurotransmitters contribute to the pathophysiology of AD. However, our data demonstrated that CV or CV-LN administration significantly decreased MDA levels while increased GSH levels, indicating strong antioxidant effects. These outcomes are consistent with previous research by Abu-Serie et al.^66^ and are attributed to the rich content of antioxidant phytochemicals in CV, particularly terpenoids, carotenoids, tocopherols, polyphenols, chlorophyll, and the trace element selenium ^6^.

In the current investigation, phosphorylated Tau (p-Tau) and Aβ1-42 levels were markedly higher in the cortex and hippocampus of AlCl_3_-induced AD-like rats. Amyloid precursor protein (APP) is broken down by β-secretase (β-site APP cleaving enzyme; BACE-1) into the soluble sAPPβ and C99 peptide, which is then cleaved by γ-secretase to produce Aβ-40 and Aβ-42^67^. Accumulation of Aβ contributes to neurodegeneration through plaque growth, Tau hyperphosphorylation, NFTs, and neuroinflammation^68^. In AD, there is a suppression of the phosphatidylinositol 3-kinase (PI3K)/Akt pathway and over activation of its downstream kinase GSK3β which contributes to Aβ toxicity and neuroinflammation^69^. According to Fronza et al.^70^, Tau hyperphosphorylation and cognitive impairment in AD are caused by GSK3β. Its overexpression also impacts the metabolism of acetylcholine, which leads to cholinergic deficiencies in AD. Therefore, targeting the Aβ synthesis and PI3K-Akt/GSK3β axis are essential for treating AD. AlCl₃ inhibits PI3K/pAkt^71^, while CV reverses this effect^72^. In the current study, CV or CV-LN administration significantly corrected the elevated Aβ levels, P-Tau expression and GSK3β activity caused by aluminum exposure. Therefore, CV or CV-LN offer neuroprotection via the upregulation of PI3K-Akt axis and downregulation of GSK3β which promote Aβ clearance and Tau dephosphorylation.

Silent information regulator 1 (SIRT1), a NAD⁺-dependent class III histone deacetylase, is essential for energy metabolism, transcription control, and physiological processes linked to aging^73^. By deacetylating proteins such as FOXO3a, and NF-κB, SIRT1 inhibits oxidative stress, neuroinflammation, and apoptosis, hence exerting neuroprotective benefits^74,75^. In the present study, the brain tissues of AD rats showed a significant down regulation of SIRT1 levels in contrast to controls. According to Zhu et al.^76^, Aβ accumulation blocked SIRT1 in AD animal models, while betaine and resveratrol drastically decreased Aβ deposition, and enhanced memory in the brain by activating SIRT1^77^. Active SIRT1 reduces the amount of Aβ in AD brain by raising the rate of α-secretase^77^ and suppressing the actions of β-secretase^78^ and GSK3β^79^. Aβ accumulation leads to mitochondrial dysfunction and increases the levels of ROS in brain, which triggers β secretase, and enhances further accumulation of Aβ, creating a vicious cycle^80^. In addition, SIRT1 prevents tauopathy by deacetylating tau protein in diabetic models^81^. According to our findings, the orally administered CV and CV-LN groups showed increased levels of GSH and SIRT1 and lowered levels of Aβ protein in the hippocampus and cerebral cortex, indicating that CV as a SIRT1 activator can lessen the Aβ-induced oxidative brain damage.

Brain-derived neurotrophic factor (BDNF) is involved in neuronal growth and plasticity changes related to memory^82^. Reduced production of BDNF in AD brains implies the progression of the disease and is mediated by the tropomyosin receptor kinase B (TrkB)/ cyclic AMP response element-binding protein (CREB) signaling pathway^83^. While over expression of BDNF leads to Tau dephosphorylation via activation of TrkB and PI3K signaling^84^. AlCl_3_ exposure reduced BDNF expression^49,85^ and promotes Aβ plaque accumulation by disrupting axonal transport and synaptic function. Additionally, SIRT1 regulates miRNA-134 pathway by increasing CREB and BDNF expressions via methyl-CPG binding protein 2 (MeCP2) acetylation^86^ and a Yin Yang 1 (YY1) -containing repressor complex^87^. Key signaling pathways (CREB, BDNF, Nrf2, PI3K) involved in synaptic plasticity, cognitive function, and neuroprotection are disrupted by dysregulation of miRNA-134, which also targets SIRT1 mRNA^87^. Thus, activating SIRT1 and inhibiting miRNA-134 will then restore the downstream CREB/BDNF expression and offer neuroprotection.

In the present study, decreased BDNF levels and poor synaptic plasticity were caused by increased miRNA-134 and decreased SIRT1 expression in AlCl_3_-induced AD-like rat hippocampus neurons. These effects were reversed by CV or CV-LN treatment, which improve synaptic function and BDNF expression (Table 3), confirming the important role of CV and CV-LN in the modulation of the SIRT1/miRNA-134/GSK3β axis in AD. Similarly, Huang et al.^88^ also showed that the upregulating CREB, BDNF, BCL2, and the inhibiting miRNA-134 can lessen the damage caused by cerebral ischemia. Furthermore, improved formulation of CV-LN showed better results than free CV in modulating the amyloidogenic and SIRT1/miRNA-134/GSK3β pathways. This is probably because of its increased bioavailability, prolonged release, and targeted brain delivery, which may highlight its potential for mitigating aluminum- induced neurodegeneration and AD.

Neuroinflammation in AD is primarily mediated by astrocytes and microglia. Elevated glial fibrillary acidic protein (GFAP) expression^89^ indicates astrocyte activation, whereas Iba-1 expression indicates microglial activation^90^. Aβ plays a central role in this process because it stimulates both cell types, causes pro-inflammatory cytokines to be released continuously, exacerbates further Aβ aggregation, and speeds up the progression of AD^91^. Increased levels of GFAP in the current investigation are in line with Justin-Thenmozhi et al.^46^ and indicate inflammation and astrocytic activation in AlCl_3_-induced AD-like rats (Table 3). However, this inflammatory cascade was stopped by treatment with CV or CV-LN, which prevented astrocyte activation in hippocampus tissues.

Programmed cell death, or apoptosis, occurs through both extrinsic and intrinsic routes. Mitochondrial dysfunction leads to cytochrome c leakage, controlling mitochondrial signaling in the intrinsic route. This process is regulated by the pro-apoptotic protein BAX and the anti-apoptotic BCL2. BAX promotes apoptosis by triggering caspase-3, a crucial effector enzyme in apoptotic cascades, whereas BCL2 prevents this process^92^. Additionally, neuronal apoptosis is modulated by Akt /GSK3β signaling pathway. Caspase-3 promotes Tau hyperphosphorylation by the cleavage of Akt, which in turn activates GSK3β, and the production of toxic APP fragments^93^. Oxidative stress and inflammation, both exacerbated by AlCl_3_ exposure, are major triggers of caspase-3-mediated neuronal apoptosis^3^. Al decreases Akt/GSK-3β in the hippocampus and cortex^46^. Our results of elevated BAX, BAX/BCL2 ratio and caspase-3 expression levels and lowered BCL2 expression levels were observed in AlCl_3_-exposed rats, indicating neuronal apoptosis and aligned with Justin-Thenmozhi et al.^46^ and Ekundayo et al.^94^ On contrast, CV or CV-LN supplementation has been demonstrated to increase BCL2 and decrease caspase-3, BAX expression levels and BAX/BCL2 ratio as compared to AlCl_3_-induced AD-like group (Fig. 4), hence supporting its anti-apoptotic function. This outcome is consistent with earlier research findings^95–97^. This antiapoptotic activity of CV might be ascribed to its antioxidant properties, with the activation of SIRT1 and Akt/GSK3β signaling most likely mediating decreases in Tau phosphorylation and GSK3β activity. The best effect on BAX and BCL2 was achieved by invasomal formulations owing to improved bioavailability and targeting.

The present findings showed that AlCl_3_-induced AD-like group displayed signs of neurotoxicity, cognitive decline, and cell death, which were corroborated by the histological pictures of tissue damage, including extensive degenerative changes and shrinkage in neurons and neuroglia cells with disrupted hippocampal cell layers, accumulation of acidophilic amyloid materials; and irregular myelinated nerve fibers (Fig. 5). These observations are in line with earlier studies by Haider et al.^98^ and Sumathi et al.^99^. However, these symptoms were markedly improved by CV and particularly CV-LN treatments, as evidenced by changes in tissue structure, biochemical and behavioral results which are attributed mainly to antioxidant, anti-inflammatory, and antiapoptotic properties of CV.

The neuroprotective effects of CV are primarily mediated through its rich composition of antioxidant and anti-inflammatory compounds, including astaxanthin, lutein, β-carotene, phenolic acids, flavonoids, unsaturated fatty acids, and trace minerals (Cu, Zn, Se, Fe)^6^ as confirmed through HPLC^66^, and Gas chromatography–mass spectrometry (GC/MS)^100^ analyses. The presence of hydroxyl groups and unsaturated bonds in its bioactive compounds facilitates effective free radical scavenging and metal ions chelation to prevent radical generation. The bioactive compounds present in CV contribute to neuroprotection through a multi-step mechanism. They enhance the activity of endogenous antioxidant enzymes, thereby reducing lipid peroxidation, oxidative stress and inflammation, which are key contributors to neurodegeneration. This antioxidant effect leads to the activation of SIRT1 which subsequently downregulates miRNA-134 expression, resulting in increased BDNF levels and improved synaptic function. SIRT1 further modulates amyloidogenic pathways by enhancing α-secretase activity and inhibiting β-secretase, reducing amyloid-β (Aβ) accumulation. Concurrently, SIRT1 inhibits GSK3β activity, preventing tau hyperphosphorylation. These actions collectively reduce Aβ and tau pathology, promote neuronal survival, and improve cognitive function, highlighting the prospective probable therapeutic potential of CV and its niosomal formulation in neurodegenerative disorders such as Alzheimer’s disease.

### Limitations and recommendations for future work

This study shows promising neuroprotective effects of CV and CV-LN, but several limitations remain. The rat model and high AlCl₃ dose may not reflect human conditions, and long-term safety is uncertain due to the lack of chronic toxicity assessments. Dose optimization and direct mechanistic validation of the proposed SIRT1/miRNA-134/GSK3β pathway were not conducted. Pharmacokinetic profile including brain permeability, systemic bioavailability, release behavior, and entrapment efficiency studies are also missing. To address these limitations, future research will include chronic toxicity evaluations, dose optimization, human mimic-exposure models, mechanistic validation using gene knockdown strategies or specific pathway inhibitors, pharmacokinetic profiling, and nuclear magnetic resonance (NMR)-based characterization to better support the clinical potential of CV-LN.

## Conclusion

The current study demonstrated that CV and particularly its novel nano-formulation (CV-LN) offer strong neuroprotective and memory-enhancing effects against aluminum-induced neurotoxicity, primarily via modulation of the SIRT1/miRNA-134/GSK3β axis. CV and CV-LN contribute to modulating neurotransmitter balance, improving synaptic function, strengthening antioxidant defenses, preventing amyloid plaque accumulation and Tau protein hyperphosphorylation, and minimizing neuroinflammation and neuronal cell death which are also supported by the recovery of the hippocampus tissue structure. Notably, CV-LN exhibited superior efficacy, possibly due to improved brain permeability and bioavailability. These findings suggest that CV and particularly CV-LN hold promise as therapeutic candidates for managing neurodegenerative conditions such as AD. This innovative approach paves the way for future research assessing long-term safety, optimizing dosing, validating mechanisms, and conducting pharmacokinetic and molecular analyses to confirm clinical relevance.

## Supplementary Information

Below is the link to the electronic supplementary material.

Supplementary Material 1

Supplementary Material 2

## Data Availability

The data used to support the findings of this study are available from the corresponding author upon request.
