# Supplementary material for: Neuroprotective effects of Chlorella vulgaris loaded niosomes via SIRT1 activation in aluminum chloride-induced Alzheimer’s model
Source: Sci Rep. 2025 Nov 18;15:40361. doi: 10.1038/s41598-025-25892-7 (PMC12627090; doi:10.1038/s41598-025-25892-7)
Supplement: Supplementary file 1 — Supplementary Material 1 [file 41598_2025_25892_MOESM1_ESM.pdf]

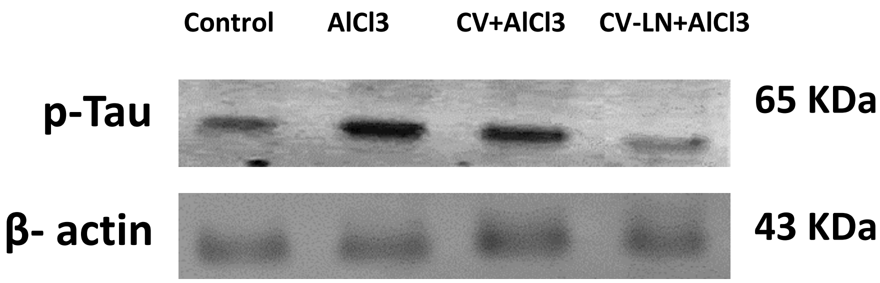

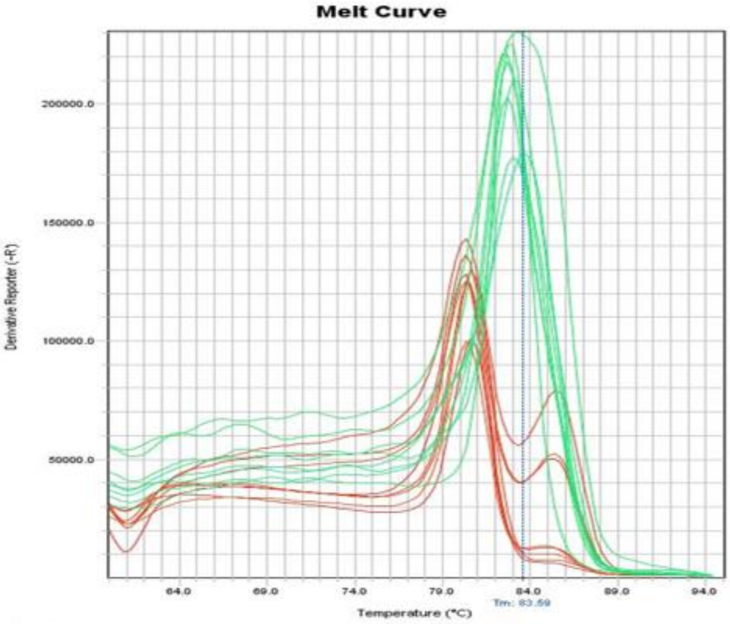

The melt curve is presenting the replicates of the samples and their normalization versus the house keeping gene
